# Probing Radical‐Induced Magnetic Moment Modulation at Cobalt Interfaces via Soft X‐Ray Photoelectron Spectroscopy

**DOI:** 10.1002/chem.202503436

**Published:** 2026-02-25

**Authors:** Maria Benedetta Casu

**Affiliations:** ^1^ Institute of Physical and Theoretical Chemistry University of Tuebingen Tuebingen Germany

**Keywords:** cobalt, interface, photoemission, radical thin films, soft X‐rays

## Abstract

We investigate the influence of the deposition of a Blatter‐pyr radical layer on polycrystalline cobalt using X‐ray photoelectron spectroscopy (XPS), treating the full interface as a hybrid system. Our analysis reveals a chemical bond between the radical layer and cobalt surface, resulting in the loss of radical character upon deposition. Despite this, the chemisorption process effectively modulates the cobalt magnetic moment, which decreases to 1.53 μ_B_, consistent with recent findings in organic closed‐cell systems. These results highlight the sensitivity of XPS in detecting subtle electronic and chemical changes at metal/radical interfaces and emphasize the role of interfacial bonding in tuning magnetic properties. While the study focuses on interfacial magnetism rather than preserving molecular spin, future strategies may involve designing molecules with selective anchoring groups to maintain radical integrity. Measurements were conducted at room temperature without external magnetic fields, underscoring the practicability of the approach. Given the experimental challenges in probing such hybrid systems, our method offers a versatile and accessible pathway for identifying organic/ferromagnetic interfaces with promising spintronic potential.

## Graphical abstract

X‐ray photoelectron spectroscopy reveals to be a powerful technique to explore subtle changes of the electronic structure at the radical/cobalt interface. The radical chemisorption influences the cobalt electronic structure and consequently its magnetic properties. The method enables the preselection of organic/ferromagnetic and radical/ferromagnetic interfaces with high potential for spintronics in a relatively straightforward and simple way.

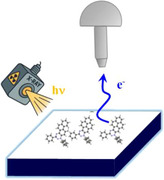

## 1 Introduction

“The interface is the device” is a sentence that became famous after the Nobel Prize lecture given by Herbert Kroemer in 2000. In its simplicity, the sentence underlines the complexity of connecting two materials and merging their properties, also willing to use them for applications. In fact, not only are interfaces extensively investigated, but many of them are at the core of the technological development driven by electronics through the extreme miniaturization of new devices.

Organic/metal interfaces have been the focus of intense investigations for at least three decades [1, 2, 3, 4, 5]. This opened the way to consider the organic/ferromagnetic interfaces as potential components of new devices [6]. A variety of effects, depending on the strength of the interaction between the molecules and the inorganic surface are emerging in terms of modifications of the spin properties of the inorganic materials at the interface when compared to the bulk [7, 8, 9, 10, 11]. The organic layer at an organic/ferromagnetic metal interface often acquires a spin polarization because of the chemical bond with the ferromagnetic film [12, 13]. In turn, the electronic and magnetic properties of the ferromagnetic film are also significantly modified [14, 15]. In addition to the prototypical fullerene (C_60_)/cobalt interface several other organic nonmagnetic materials have been recently shown to influence the magnetic properties of the cobalt suggesting the presence of a highly anisotropic layer localized at the interface.

In this work, taking advantage of the surface sensitivity of soft X‐ray techniques such as X‐ray Photoelectron Spectroscopy (XPS), we discuss the results obtained by depositing a purely organic radical layer, that is, a layer of molecules carrying a magnetic moment [16, 17, 18, 19], on polycrystalline cobalt and identifying its impact on the properties of the metallic surface. In the polycrystalline form, cobalt maintains its magnetic properties [20, 21, 22] and it is broadly used in spintronic devices. Blatter‐pyr, obtained by fusing a Blatter radical to a pyrene unit, is a chemically and thermodynamically stable purely organic radical [23, 24] that can be evaporated in ultra‐high vacuum (UHV), under controlled conditions [25]. It is a well‐characterized radical derivative [19, 25, 26], therefore, it is a convenient choice to grow the radical layer on the cobalt surface.

## 2 Concepts

XPS spectra are characterized by high‐intensity features that belong to core‐level photoemission effects [27, 28]. While XPS is a well‐established technique for probing the electronic structure of materials, its utility extends far beyond this primary application. Due to its high surface sensitivity and elemental specificity, XPS enables the quantitative determination of the stoichiometry of analyzed systems [27, 29]. Moreover, it provides detailed insights into the local chemical environment of constituent elements, facilitating the identification of chemical bonding configurations and charge transfer processes at surfaces and interfaces. The integrated area of the main lines corresponding to photoelectrons emitted from a given element, together with their satellites, is proportional to the concentration of that same element in the investigated system. In highly resolved XPS spectra, the rich structure allows fitting the lines including contributions from different atomic sites of the same element which, due to a different chemical environment, are expected to show different binding energies [30, 31]. With its expanded analytical capabilities, XPS has recently become an important tool for exploring materials in emerging quantum technologies [32, 33].

XPS is also instrumental in assessing the stability of thin films, particularly under conditions such as prolonged X‐ray irradiation or ambient air exposure, thereby offering valuable information on post‐deposition modifications [25, 34, 35, 36]. Furthermore, we previously demonstrated that the technique is exceptionally well‐suited for the characterization of radical thin films and assemblies, especially when these are deposited under controlled evaporation conditions [25, 26, 37, 38]. We demonstrated that XPS, when combined with a rigorous and reproducible fitting protocol, enables the reliable identification and quantification of the radical character in such films, yielding results in excellent agreement with complementary analytical methods, such as electron paramagnetic resonance (EPR) spectroscopy that is the technique typically used for radical characterization [25, 26, 38, 39, 40, 41, 42, 43, 44, 45].

The fitting procedure employed in XPS for investigating radical thin films is guided by rigorous chemical and physical principles rather than by purely mathematical optimization. Spectral features are modeled using Voigt profiles, which represent the convolution of Gaussian and Lorentzian components. This approach reflects the distinct physical origins of line broadening: intrinsic effects such as core‐hole lifetime broadening, vibronic coupling, and inhomogeneous broadening contribute to the Lorentzian component, while instrumental factors, such as analyzer resolution, X‐ray source bandwidth, and sample heterogeneity, manifest as Gaussian broadening.

The intrinsic Lorentzian width is linked to the core‐hole lifetime via the Heisenberg uncertainty principle. For instance, in organic materials, the Lorentzian full width at half maximum (FWHM) is typically around 80 meV for the C 1s orbital and approximately 100 meV for the N 1s orbital [30]. The Gaussian contribution, on the other hand, arises from the experimental setup and is assumed to follow a normal distribution due to the aforementioned instrumental limitations.

Our fitting methodology is adapted from protocols established for closed‐shell molecular systems [38, 46]. The final spectral fit is achieved through a series of self‐consistent iterations, each incorporating progressively refined physical and chemical constraints. This iterative approach minimizes parameter interdependence, with final dependency values approaching zero in the present study.

Constraints are imposed based on elemental concentrations and binding energy values, which must conform to known electronegativity trends and literature‐reported data. Core‐hole lifetimes are used to define the Lorentzian widths for each element. Importantly, the fitting protocol is designed to be universally applicable across all spectra analyzed, ensuring consistency and reproducibility.

Unlike our previous approach, which examined the properties of thin radical assemblies or layers deposited on an inorganic substrate, carefully selected to interact weakly with the radical and thereby preserve its character, this work investigates the radical/cobalt interface, analyzing both materials in detail and exploring how the nature of their interaction influences the properties of the interfacial hybrid system.

We further illustrate how X‐ray photoemission enables relatively straightforward quantification of changes in the magnetic moment of cobalt atoms at the interface with a radical. Notably, this analysis is performed at room temperature and does not require the application of external magnetic fields. This highlights the sensitivity and versatility of the technique, as it allows for the prediction of subtle magnetic variations that arise from interfacial interactions and chemical modifications.

## 3 Results and Discussion

**FIGURE 1:**
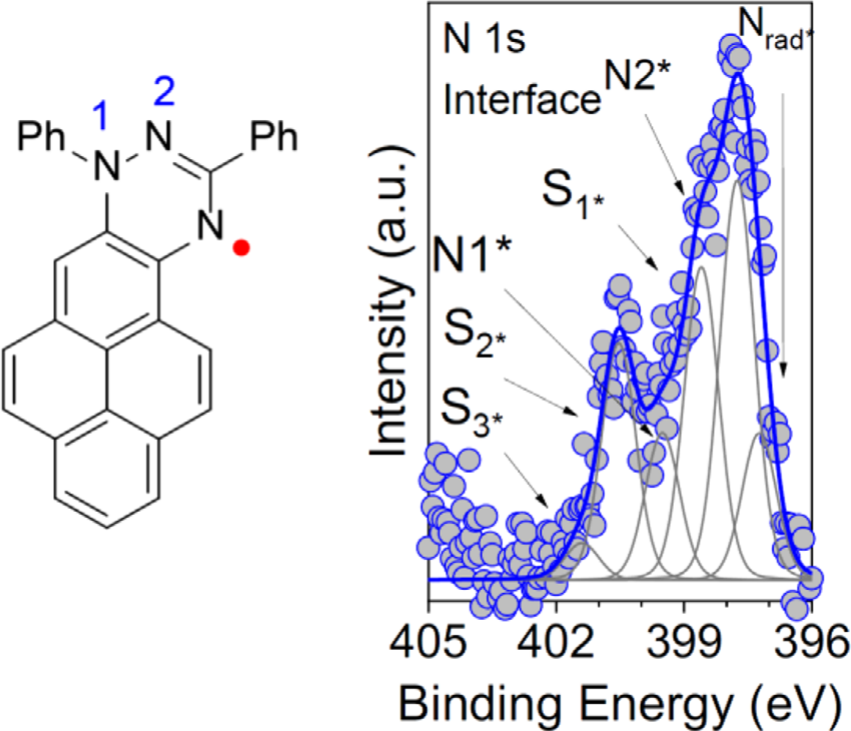
Blatter‐pyr films deposited on polycrystalline cobalt. N 1s core‐level spectrum of an interfacial layer (0.4 nm) spectrum, together with its fit components, named as shown in the Blatter‐pyr molecular structure (left panel, Ph stands for the phenyl ring). The asterisks identify the interfacial nature of the features. See the Supporting Information for the fit parameters.

In this work, we focused on the N 1s core‐level spectra that provide insights into the magnetic behavior of the Blatter‐pyr radical, as the unpaired electron is predominantly delocalized over the triazine ring (Figure 1). The spectra exhibit three distinct main peaks, corresponding to the three chemically inequivalent nitrogen atoms (Figure 1). In thick films, where the interfacial effects are not visible, i.e., beyond the limit of the XPS sampling depth [47], the peak near 399.0 eV is attributed to the pyridine‐like nitrogen (N2), which is bonded to both a carbon and a nitrogen atom. The peak at 400.9 eV is assigned to the nitrogen also connected to the phenyl ring (N1). Due to the electron delocalization, the peak at lower binding energy (398.2 eV) is associated with photoemission from the nitrogen radical site (Nrad) [25, 26].

The N 1s core level spectra of the interfacial layer (i.e., the first organic layer formed by the molecule directly in contact with the metallic ferromagnet) are characterized by very broad features, different from those characterizing the thick films, and by a shift of around 1 eV towards lower binding energies (Figure 1) [19, 25, 26]. This behavior indicates a strong interaction, of a chemical nature, between the Blatter‐pyr and the cobalt substrate [4, 48, 49, 50, 51, 52]. Also, the intensity of the satellite features is higher. This is a further robust indication of the strong chemisorption of the Blatter‐pyr on cobalt [31, 49].

However, this interaction has a strong impact on the radical character of the interfacial molecular layer. In films of intact molecules, the radical character is linked to the presence of the feature at lower binding energy (N_rad*_ in Figure 1) and correlates with its intensity [25, 26, 34]. This feature exhibits very low intensity in the N 1s core‐level spectrum of the interfacial film, indicating only a residual radical character. Such attenuation reflects a substantial alteration of the chemical environment of the molecules adsorbed directly onto the cobalt surface, as compared to those embedded within the bulk of the radical film. This difference arises from the formation of chemical bonds between the cobalt atoms and the Blatter‐pyr radical, which modifies the electronic structure, and quenches the radical character at the interface. The observed spectral changes suggest that the chemisorption process directly involves the pristine unpaired electrons of the Blatter‐pyr system [53] (Figure 1, the interfacial features are indicated with an asterisk to underline their different chemical environment with respect to thick films).

X‐ray absorption spectroscopy experiments revealed a high degree of azimuthal disorder in the interfacial layer, further supported by electronic structure calculations based on Density Functional Theory (DFT) [53]. The calculations show that different radical/Co geometries are possible (for example, the pyrene unit is oriented along the Co[1010] direction (or parallel to the Co [0110] direction) and one of the phenyl rings protrudes out of the surface) with relatively close binding energies that may influence the XPS line broadening of the N 1s curves of the interfacial layer [53]. Note that the XPS signals in this work are averaged over the area of the illuminated spot on the sample surfaces, because standard XPS is not laterally resolved.

This finding offers a comprehensive insight into the interfacial processes that affect the radical character of the first molecular layer deposited on cobalt. It now becomes a logical step to ask whether the strong interaction with the radical also alters the electronic and magnetic properties of the interfacial cobalt itself. The element‐sensitivity characterizing XPS allows us to investigate simultaneously the photoemission from core level states from the molecular and the cobalt side of the interface, and, thus, to answer this question.

Recent studies have suggested that a layer of C_60_ on platinum dramatically increases the anisotropic magnetoresistance via the metal molecule orbital hybridization and predict a significant spin‐orbit coupling through the Pt layer, concomitant with a change in the metal magnetic moment [54].

**FIGURE 2:**
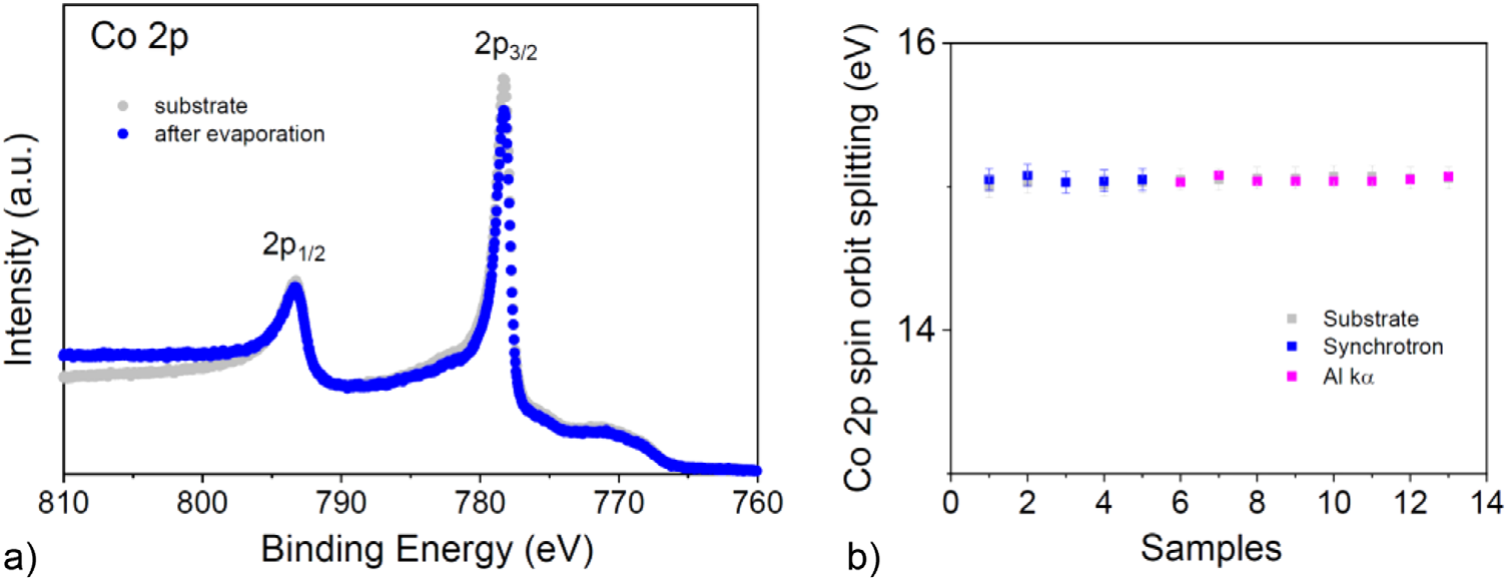
(a) Typical Co 2p core‐level spectra before and after evaporation (hν = 1486.8 eV). (b) Co 2p_3/2_ – Co 2p_1/2_ binding energy separation for a set of the investigated cobalt surfaces before (grey) and after deposition of the radical layer for two different photon energies (at synchrotron hν = 990 eV and hν(Al kα) = 1486.8 eV).

The rate of electronic transitions observed in XPS is governed by the Fermi Golden Rule, which defines that transition probabilities depend on both the initial and final electronic states. Among the initial state effects, spin‐orbit coupling plays a significant role, particularly in shaping the spectral features associated with core‐level photoemission from orbitals such as p, d, and f, which exhibit spin‐orbit splitting. In this context, we aimed to investigate whether XPS can mirror the influence on the spin‐orbit splitting at the radical/cobalt interface due to the orbital hybridization between the molecular and the metallic orbitals, induced by chemisorption. To probe this assumption, we employed XPS and focused specifically on the Co 2p core‐level spectra, comparing measurements taken before and after deposition of the radical layer. Co 2p core level spectra are characterized by the presence of a doublet because of the mentioned spin‐orbit coupling (Figure 2). Figure 2b shows their binding energy separation for a set of samples, before and after evaporation of a Blatter‐pyr radical layer. The spectra have been collected using synchrotron radiation (hν = 990 eV, inelastic mean free path (λ) = 1.4 nm, more surface sensitive) and in the lab with a monochromatized Al Ka X‐ray source (hν = 1486.8 eV, *λ* = 2.5 nm, more bulk sensitive). Changing photon energy allows tuning the kinetic energy of the collected photoelectrons, that is, their inelastic mean free path, resulting in more surface sensitive measurements at 990 eV.

The measured separation of 15.05 ± 0.01 eV aligns with established literature values [55]. This energy difference remains invariant upon deposition of the radical layer and across varying photon energies, indicating that the Co 2p levels are too deep in energy to be perturbated by the different chemical environment at the interface. Therefore, in the framework of this study, we were unable to prove the correlation between hybridization and enhanced spin‐orbit coupling by investigating the Co 2p core level spectra.

Considering these results, one promising approach that we intend to explore in the future is the investigation of the Co 3p core‐level spectra. However, these have a binding energy of approximately 60 eV, placing them within the valence band range. This makes their analysis particularly challenging, not only due to their spectral overlap but also because fitting procedures for these spectra are scarcely discussed in literature.

**FIGURE 3:**
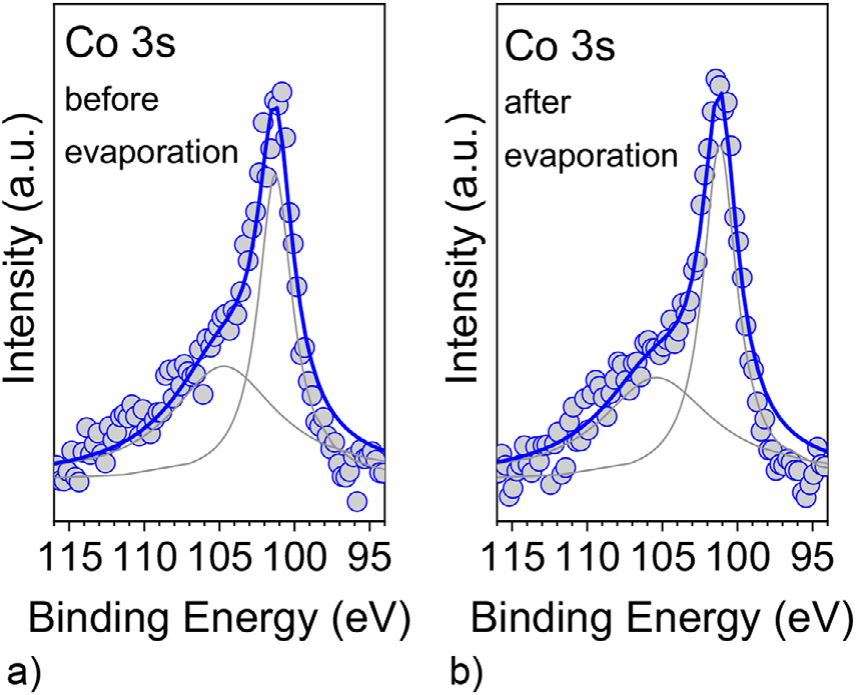
Co 3s core‐level spectra (a) before and (b) after evaporation of the interfacial radical layer, together with their fit. See the Supporting Information for the fit parameters.

To fully harness the information embedded within the XPS spectrum, we focused our analysis on the Co 3s core‐level region. The 3s core‐level photoemission spectra in transition metals have a complex structure. They are characterized by the multiplet splitting that may occur when unpaired electrons occupy valence states [56]. The Co 3s core‐level spectra are characterized by a doublet. We fitted the spectra before and after the deposition of the interfacial film, by using a Doniach–Sunjic line shape (Figure 3) [56]. We find that the doublet binding energy separation is around 3.5 eV, in agreement with the literature for polycrystalline cobalt and cobalt compounds [57]. The energy separation between the two peaks correlates with the 3s‐3d exchange interaction energy, while the peak intensities of the doublet features correlate with the total spin and, thus, with the magnetic moment [56, 58]. We calculated the magnetic moment, obtaining 1.78 μ_B_ and 1.53 μ_B_ before and after evaporation. Recent studies found a correlated random‐anisotropy field in hybrid Co/molecule bilayers induced at the cobalt surface by the hybridization with the molecules [59]. The coexistence of multiple molecular conformations, also reported for the present system [53] leads to a random net anisotropy with a characteristic length scale set by the molecular dimensions. We can infer that a similar phenomenon occurs also for the Blatter‐pyr radical/polycrystalline cobalt interface. Also in this case, it is useful to consider that XPS signals are averaged over the area of the illuminated spot on the sample surface. Consequently, the calculated magnetic moment is likewise averaged over the same surface area.

Here, we would like to mention that this method has yielded reliable values for the magnetic moment in cobalt‐based materials, chromium compounds, and iron alloys [56, 57, 58, 60, 61, 62, 63]. Some critical issues have been reported in other cases, such as in pure iron [64, 65, 66]. This is because the 3s satellites can generally be interpreted through exchange splitting, but the 3s splitting is not universally proportional to the magnetic moments across all materials. Only when the charge‐transfer satellite in the 2p spectra is negligible, the 3s splitting reflects the local moment of the ground state [67]. This explains why the method provides reliable results in the present study, which are also consistent with *ab initio* calculations [53].

## 4 Summary and Outlook

We have investigated the influence of a Blatter‐pyr layer deposited on polycrystalline cobalt by using the full capability offered by X‐ray photoelectron spectroscopy, focusing on the interface as a functional system. We have found that the radical and the cobalt are chemically bound at the interface. This has strong implications for both materials. On the one hand, Blatter‐pyr loses its radical character upon deposition, on the other its chemisorption is a viable way to influence the magnetic properties of the cobalt layer. We have found that the cobalt magnetic moment decreases to 1.53 μ_B_ after evaporation of a single radical layer, analogously to what has been recently observed for organic close cell systems [54, 68, 69, 70, 71]. These findings underscore the sensitivity of XPS in detecting subtle electronic and chemical modifications at metal/radical interfaces and highlight the importance of chemical bonds in tailoring the magnetic and electronic properties at the interface.

The loss of radical character does not undermine the current investigation, which focuses on probing interfacial magnetic behavior rather than maintaining the radical spin integrity. However, future studies could benefit from having intact radicals on cobalt surfaces. This may be accomplished by engineering molecular structures with functional groups that preferentially bind to the ferromagnetic surfaces, effectively inhibiting chemisorption of the radical unit, an approach that has been successfully demonstrated on gold surfaces [36, 72, 73, 74, 75, 76].

Our study highlights that predictive insights into the magnetic role of the organic interfacial layer can be achieved by a thorough understanding of X‐ray photoemission spectroscopy. Indeed, because magnetic properties stem directly from specific electronic structures, gaining detailed insight into the occupied states through XPS, considering also the presence of a core‐hole, can provide valuable understanding of these properties. Notably, the measurements are performed at room‐temperature and without applying an external field to the system under investigation. Work is further needed to develop the discussion and the fit procedure of Co 3p core‐level spectra that may lead to the direct correlation between the changes in the magnetic moment and an enhanced spin‐orbit coupling. Despite the relevance of such hybrid interfaces for spintronic applications, the number of organic materials known to modulate cobalt's magnetic moment at the interface remains limited. This is largely due to the experimental complexity involved, requiring a combination of high‐resolution techniques, surface sensitivity, and often extreme conditions such as high magnetic fields and low temperatures. In this context, the versatility of our approach is particularly valuable, enabling the preselection of organic/ferromagnetic and radical/ferromagnetic interfaces with high potential for spintronic functionality in a relatively straightforward and simple way.

## 5 Experimental Section

The in situ sample preparation and XPS measurements were carried out in a multichamber XPS station. A polycrystalline cobalt foil (MaTeck, 99,9% purity) was used as a transition metal surface. It was cleaned by repeated cycles of argon sputtering (2 kV) and annealing at around 573 K. The cleanliness of the surface was checked by XPS. Blatter‐pyr has been synthesized as described in Ref. [25]. The radical thin layers were evaporated by Organic Molecular Beam Deposition (OMBD, evaporation rate: 0.1–0.6 nm/min). The evaporation rate was estimated by using a quartz microbalance, and the film thickness was cross‐checked by using the attenuation of the substrate Co 2p photoemission line upon radical deposition. The XPS measurements were performed in the measuring chamber equipped with a monochromatic Al Kα source (SPECS Focus 500) and a SPECS Phoibos 150 MCD hemispherical electron analyzer. The survey spectra were recorded at 50 eV pass energy (energy resolution was 0.4 eV, energy step 0.1 eV) and the high‐resolution spectra at 20 eV pass energy (energy step: 0.05 eV). The XPS binding energies were calibrated by setting the Co 2p signal at 778.3 eV [77].

Photon‐dependent XPS measurements were conducted at the LowDosePES end‐station at the PM4 beamline (*E*/Δ*E* = 6000 at 400 eV) of the BESSY II synchrotron facility in Berlin [78, 79]. The end‐station was a multichamber set‐up with a separate preparation chamber, used for sputtering and annealing the cobalt substrate and for film deposition. The measuring chamber (base pressures 4 × 10^−10^ mbar) was equipped with a Time‐of‐Flight Scienta ArTOF‐10k electron energy analyzer. The film evaporation was done using the same calibrated cells as in the above‐described XPS experiments.

During all experiments, the stoichiometry of the radical films is proved by using a well‐established fit routine [25, 36, 40], systematically correlated with electron paramagnetic resonance (EPR) results on a variety of different radicals [26, 38, 40, 43, 44, 45].

All photoemission measurements were performed in normal emission. The measurements were performed on freshly prepared films. All samples were carefully monitored to avoid radiation damage during beam exposure.

The information depth in XPS was calculated according to Ref. [53]. To calculate the magnetic moment from the XPS Co 3s spectra, we used the relationship: I(S+1/2)/I(S−1/2) = (S+1)/S where I are the intensities of the doublets obtained from the fits and S is the total spins of the unpaired electrons [56]. The spectroscopic lines were fitted by using a Doniach–Sunjic line shape [56]. The magnetic moment was calculated using the equation $\mu =2\mu_{B}\sqrt{(S+1)S}$ [56, 63]. The fitting strategy was based on a well‐defined physical and chemical model, which imposes constraints on the mathematical form of the fit. These constraints assure that the resulting values retain physical significance and minimize dependency. The obtained results were further validated through comparison with the published literature values, providing reliability. The procedure was systematically applied across large sets of samples, allowing us to verify the robustness and reproducibility of the fitting procedure for each individual measurement. As a result of this approach, the binding energies obtained by fitting the curves have high accuracy, and the numerical values have two significant decimal places. This level of precision enables the identification of binding‐energy shifts below the instrumental resolution [80, 81]. Additionally, the constraints on the binding energy separation between the doublets impose constraints on their intensities minimizing the dependency values that are kept as close as possible to zero all over the present work.

## Conflicts of Interest

The author declares no conflicts of interest.

## Supporting information



Additional Supporting Information can be found online in the Supporting Information section.
The author has cited additional references within the Supporting Information [36, 38, 46].

## Data Availability

The data that support the findings of this study are available from the corresponding author upon reasonable request.
