# Supplementary material for: Probing Radical‐Induced Magnetic Moment Modulation at Cobalt Interfaces via Soft X‐Ray Photoelectron Spectroscopy
Source: Chemistry. 2026 Feb 25;32(18):e03436. doi: 10.1002/chem.202503436 (PMC13174908; doi:10.1002/chem.202503436)
Supplement: Supplementary file 1 — Additional Supporting Information can be found online in the Supporting Information section. The author has cited additional references within the Supporting Information [36, 38, 46]. [file CHEM-32-e03436-s001.docx]

Supporting Information

Probing Radical-Induced Magnetic Moment Modulation at Cobalt Interfaces via Soft X-Ray Photoelectron Spectroscopy

Maria Benedetta Casu*^[a]^

[a] Prof. Dr. M. B. Casu
Institute of Physical and Theoretical Chemistry
University of Tuebingen
Auf der Morgenstelle 18
D-72076 Tuebingen
E-mail: benedetta.casu@uni-tuebingen.de

Table of contents

1) Fit parameters for the interfacial N 1s core level spectra.

2) Fit parameters for the XPS Co 3s core level spectra.

3) References

1. Fit parameters for the N 1s spectra of an interfacial Blatter-pyr layer as in Figure 1.

A detailed description of the fit procedure used for the radicals is given in Ref. ^[1]^, and a general approach for fitting XPS spectra of organic thin films is also described in Ref ^[2, 3]^.

The fit procedure systematically holds for all samples of the film/interface of a specific molecule, prepared and measured under the same conditions. In this way, we can also identify the samples that do not correspond to the expected stoichiometry. We work on sets of measured samples that are large enough to be statistically significant.

The three nitrogen peaks in the Blatter-pyr N 1s spectra are assigned to photoelectrons emitted from the nitrogen bound also to the phenyl ring (N1) and the N2 atom (a carbon atom and a nitrogen atom as neighbors), see also the main text. Because of the delocalization of the unpaired electron, the peak at lower binding energy is assigned to photoelectrons emitted from the nitrogen radical (N3=Nrad).

**Table S1.** Fit parameters for the N 1s spectra of an interfacial Blatter-pyr layer. The features are labelled according to the molecular structure shown in Figure 1.

The expected stoichiometric values for the N1s elemental analysis are:

Nradical = Nimino = Namino = 33%.

The difference between the theoretical values and the intensity values found for the interfacial layer indicate the strong interaction of chemical nature between the Blatter-pyr and the cobalt surface. Note that also the satellite features also contribute to the stoichiometric evaluation and must be considered.

|  | **Energy**  **(eV)** | **Lorentzian**  **Width (eV)** | **Gaussian**  **Width (eV)** | **Intensity**  **(%)** |
| --- | --- | --- | --- | --- |
| Nrad* | 397.2 | 0.1 | 0.9 | 11.4 |
| N2* | 397.8 | 0.1 | 0.9 | 31.1 |
| S_1*_ | 398.6 | 0.1 | 0.9 | 24.5 |
| N1* | 399.5 | 0.1 | 0.9 | 11.5 |
| S_2*_ | 400.5 | 0.1 | 0.9 | 18.6 |
| S_3*_ | 401.4 | 0.1 | 0.9 | 2.9 |

2. Fit parameters for the XPS Co 3s core level spectra.

See the main text for the details of the fitting procedure.

**Table S2.** Fit parameters for the Co 3s core level spectra as in Figure 3.

| **Substrate** | **Energy**  **(eV)** | - **(eV)** | **Asymmetry**  **parameter** | **Width**  **(eV)** |
| --- | --- | --- | --- | --- |
| Component 1 | 101.3 | 3.5 | 0.001 | 1.4 |
| Component 2 | 104.8 |  | 0.03 | 4.2 |
| **Interfacial film** |  |  |  |  |
| Component 1 | 101.2 | 4.3 | 0.001 | 1.4 |
| Component 2 | 105.5 |  | 0.03 | 4.4 |

*R^2^*= 0.93347 and *R^2^*= 0.90616 for the fit before and after evaporation.

**Table S3.** Calculated Intensity ratios and magnetic moments according to the fit parameters in Table S2.

|  | **Intensity**  **I_2_/I_1_** | **Calculated magnetic moment**  **µ_s_ (µ_B_)** |
| --- | --- | --- |
| Substrate | 0.34 | 1.78 ± 0.09 |
| Film | 0.29 | 1.53 ± 0.09 |

3) References

[1] T. Junghoefer, E. M. Nowik-Boltyk, J. A. de Sousa, E. Giangrisostomi, R. Ovsyannikov, T. Chassé, J. Veciana, M. Mas-Torrent, C. Rovira, N. Crivillers and M. B. Casu, Stability of Radical-Functionalized Gold Surfaces by Self-Assembly and on-Surface Chemistry *Chem. Sci.* **2020**, *11*, 9162-9172.

[2] S.-A. Savu, I. Biswas, L. Sorace, M. Mannini, D. Rovai, A. Caneschi, T. Chassé and M. B. Casu, Nanoscale Assembly of Paramagnetic Organic Radicals on Au(111) Single Crystals *Chem.-Eur. J.* **2013**, *19*, 3445-3450.

[3] S.-A. Savu, M. B. Casu, S. Schundelmeier, S. Abb, C. Tonshoff, H. F. Bettinger and T. Chassé, Nanoscale Assembly, Morphology and Screening Effects in Nanorods of Newly Synthesized Substituted Pentacenes *RSC Adv.* **2012**, *2*, 5112-5118.
